# The Significance of the Presence of Lactic Acid, and of Deficiency or Absence of Free Hydrochloric Acid, in the Gastric Contents as a Means of Diagnosis

**Published:** 1907-04-20

**Authors:** 


### Carcinoma of the Stomach.

### The Significance of the Presence of Lactic Acid, and of Deficiency or Absence of Free Hydrochloric Acid, in the Gastric Contents as a Means of Diagnosis.

It is a mistake to suppose that the absence of free hydrochloric acid from the contents of the stomach is by any means pathognomonic of gastric carcinoma. Very far from it. Nevertheless, clinical examination of the gastric juice for free hydrochloric acid has a certain value, and a real value, in making the diagnosis, provided the limitations of this value are clearly understood. Unfortunately, in one's student days, one hears the teaching that "the absence of hydrochloric acid from the gastric juice in this patient favours the diagnosis of carcinoma of the stomach," and one is apt to take this to mean that "when a patient has no free hydrochloric acid in his gastric juice he most probably has a gastric carcinoma." The result is that one continually comes across the notion that: "Absence of free HCl = carcinoma of the stomach."

In regard to lactic acid, in the same way, students are very apt to carry away with them the belief that the presence of lactic acid in the vomit is necessarily pathological.

Neither the absence of free hydrochloric acid nor the presence of lactic acid in abundance *need* be pathological at all. On the other hand, each of the conditions *may* be pathological; that is to say, may be the result of disease in the patient. To understand this, the main points in the physiology of ordinary gastric digestion must be clearly borne in mind.

### When Free HCl is Normally Found.

After a simple meal, consisting, perhaps, of beef and potatoes, what occurs in the healthy stomach? The food has been masticated and mixed with saliva; the ptyalin is converting the boiled starch into maltose. The presence of food evokes a flow of active gastric juice. This consists of water containing two ferments—pepsin and rennin—salts, and hydrochloric acid. The pepsin very soon begins to convert the proteid into albumoses and peptones; the rennin converts caseinogen into casein, and this in turn is converted into albumoses and peptones by the pepsin. What happens to the hydrochloric acid? Each molecule of it, as it comes into contact with a molecule of proteid, combines with the latter in a loose kind of way to form a molecular compound termed "syntonin"; as long as any molecules of proteid remain free, the hydrochloric acid which is secreted by the oxyntic cells of the glands at the cardiac end of the stomach becomes combined with some of it in this way. This is what is meant by *combined hydrochloric acid*. Only when more molecules of the acid have been secreted than there were molecules of proteid in the meal eaten will a surplus of hydrochloric acid be left free. It is clear that, even under perfectly healthy conditions, a considerable time must elapse before free hydrochloric will be present. The more proteid the meal contained, the later will it be before free hydrochloric can be expected. *After an ordinary mixed meal of medium size it is found that free hydrochloric acid is seldom present until three-quarters of an hour after eating.* During all this time the reaction of the gastric contents will be acid to litmus, because both combined hydrochloric acid and the acid sodium phosphate turn blue litmus red.

Fermentation of milk takes place, with the formation of lactic acid, so long as no hydrochloric acid is present free. When all the proteid has combined with hydrochloric acid to form syntonin, and a surplus is left free, the formation of lactic acid rapidly diminishes and ceases; the lactic acid already formed becomes absorbed, and in a healthy stomach lactic acid ceases to be found when there is plenty of free hydrochloric acid. This is to say that lactic acid is normally present at certain times, but when present at other times it is due to defective secretion of hydrochloric acid. The presence of lactic acid is only pathological when this acid is there at the wrong time; its presence then means precisely the same thing as deficiency of free hydrochloric acid.

As has been said, the time at which hydrochloric acid is to be expected after a meal depends largely on the nature of the meal and on the amount of proteid in it. This being so, it is not enough to know merely that such and such a patient's vomit contained no free hydrochloric acid; before we can say whether the absence is pathological or not we must know that the vomit contained no free hydrochloric acid *when vomiting occurred at such and such an interval after eating, the constituents and amount of the last meal being so and so.*

### The Importance of Test Meals.

It is on this account that test meals are given. The test meal may consist of what you will, but it must be known what is the proper interval after it; that the contents of the stomach, recovered either by vomiting or by a tube, ought to contain free hydrochloric acid in a healthy man. This interval is bound to vary with each variation in the amount and constituents of the meal. There are certain meals for which the interval has been worked out experimentally, and it is usual to employ one or other of these, recovering gastric contents at the appropriate interval after each. In order to begin with as empty a stomach as possible, a test *breakfast* is probably the best; the following are advocated by various authors, and occasions arise in which each in turn may be more advantageous than the other:—

1. *Ewald's Test Breakfast.*

| | |
|---|---|
| White bread ... ... ... ... | 2½ oz. |
| Weak tea, of which one-third is milk, with sugar *ad libitum* | ½ pint. |

The gastric contents should contain abundant free hydrochloric acid if recovered after one hour.

2. *Klemperer's Test Breakfast.*

| | |
|---|---|
| White bread ... ... ... ... ... | $2\frac{1}{2}$ oz. |
| Milk ... ... ... ... ... ... | 16 oz. |

The gastric contents should contain abundant free hydrochloric acid if recovered after two hours.

3. *Germain See's Test Lunch.*

| | |
|---|---|
| White bread ... ... ... ... ... | 5 oz. |
| Minced meat ... ... ... ... ... | $2\frac{1}{2}$ oz. |
| Cold water ... ... ... ... ... | $\frac{1}{2}$ pint. |

The gastric contents should contain abundant free hydrochloric acid if recovered after two hours.

4. *Riegel's Test Dinner.*

| | |
|---|---|
| Gravy soup, about ... ... ... ... | $\frac{1}{2}$ pint. |
| Beef steak ... ... ... ... ... | $2\frac{1}{2}$ oz. |
| White bread ... ... ... ... ... | $2\frac{1}{2}$ oz. |
| Water ... ... ... ... ... | $\frac{1}{2}$ pint. |

The gastric contents should contain abundance of free hydrochloric acid if recovered after three hours.

In regard to the method of recovering the stomach contents, either a stomach-tube or an emetic may be used. If the tube be chosen, the method should not be by lavage; that is to say, no additional fluid should be poured in, for the object is to obtain the gastric contents unaltered. Many patients strongly object to the tube; in such cases the administration of 30 grains of zinc sulphate as an emetic is preferable, the patient being told beforehand that he will be sick; and it is best that he should in any case remain in bed at least for the time being. It not infrequently happens that 30 grains of zinc sulphate fails to produce vomiting, in which case mechanical stimulation of the fauces may be required.

The best-known test for free hydrochloric acid is Günsberg's reagent, but in practice this is much less easy to apply than is the tropæolin test. All that is needed is a white saucer and some solution of tropæolin OO, which can be obtained through any chemist. Two drops of the tropæolin OO are smeared over the bottom of the saucer, and held before a fire until the saucer is at about body temperature and the tropæolin is quite dry. It forms a pale-orange stain. A drop of the gastric contents is then allowed to fall upon the centre of the yellow stain; if free hydrochloric acid be present, the yellow at once changes to a beautiful deep violet; this change of colour is produced by *free* hydrochloric acid, and not by either the combined hydrochloric acid, the lactic acid, or the acid salts, any or all of which render the gastric contents "acid" to the litmus test.

It is, of course, possible that the hydrochloric acid is present in too large an amount; if this be suspected, it is best to recover the stomach contents at a shorter interval after eating. For example, Ewald's test breakfast may be recovered in half an hour; it should then contain no free hydrochloric acid under normal conditions. If the tropæolin test shows free hydrochloric acid at this time, there is hyperchlorhydria, in association with which there is very liable to be gastric or duodenal ulceration.

If, on the other hand, no free hydrochloric acid is found at the proper time after the test meal, to what extent does this favour a diagnosis of gastric carcinoma? The answer to this question entails a discussion upon why carcinoma of the stomach should lead to deficiency of hydrochloric acid secretion. That it very often does so is certain, and it is on this account that the test for free hydrochloric acid has an undoubted diagnostic value. But how is it that gastric carcinoma thus alters the gastric juice? It is clear that it is seldom owing to the destruction of the oxyntic cells by the new growth; for the latter is commonly at or near the pylorus, whereas the oxyntic cells are in the glands at the cardiac end of the stomach. There are all sorts of theories upon the subject; but whether or not it may be the whole explanation from a strictly pathological point of view, the theory which is most useful from the clinical standpoint is that *it depends upon the patient being really ill.* When we are really ill all sorts of secretions suffer. It must be clearly borne in mind, however, that all sorts of other illnesses may cause absence of free hydrochloric acid from the gastric contents at the proper time after a meal. This is so, for example, in many case of acute illness, such as lobar pneumonia and typhoid fever; and in many chronic affections, such as mitral stenosis with failing compensation, cirrhosis of the liver, phthisical cachexia, the cachexia of malignant disease in which the primary growth is not in the stomach, and so on. It is only when the clinical evidence already points to the patient's disease being in his stomach that the absence of free hydrochloric acid at the proper time after a meal affords evidence in favour of a diagnosis of gastric carcinoma. Indeed one may summarise the matter shortly thus:—

1. The absence of free hydrochloric acid is normal for a certain time after each meal.

2. Its absence is only pathological when the conditions are such that the gastric contents of a healthy man would have contained free hydrochloric at the same interval after the same kind of meal.

3. The presence of lactic acid is normal after a meal containing milk, until such time as free hydrochloric acid should be present. The persistence of lactic acid for a longer period has the same clinical significance as the deficiency of the hydrochloric acid, and as the latter is the easier to test for, examination for lactic acid helps little for purposes of diagnosis.

4. The absence of free hydrochloric acid at the proper interval after food is an indication that the patient is seriously ill, and that his juices are consequently out of order; its absence only favours a diagnosis of gastric carcinoma when the other clinical evidence has narrowed the possibilities down so that the diagnosis lies between gastric ulcer or the like on the one hand, and gastric carcinoma on the other. With the former of these hydrochloric acid is likely to be present in excess, so that in a patient in whom there is a doubt as to whether the stomach lesion is simple or malignant, the absence of free hydrochloric acid from the stomach contents at the proper interval after a test meal favours the diagnosis of gastric carcinoma.

